# Coronary artery ectasia presenting with thrombus embolization and acute myocardial infarction: A case report

**DOI:** 10.1097/MD.0000000000005976

**Published:** 2017-01-27

**Authors:** Yongle Li, Chengcheng Wu, Wennan Liu

**Affiliations:** Department of Cardiology, Tianjin Medical University General Hospital, Tianjin Medical University, Tianjin, China.

**Keywords:** acute myocardial infarction, case report, coronary artery ectasia, thrombus embolization

## Abstract

**Rationale:**

Coronary artery ectasia (CAE) is characterized by an abnormal dilatation of the coronary arteries. CAE is often associated with the presence of slow coronary flow and may lead to acute myocardial infarction (AMI), even without total occlusion.

**Patient concerns and diagnosis:**

We report a case of a 24-year-old male patient with CAE suffering from AMI.

**Interventions:**

Percutaneous coronary intervention with aspiration thrombectomy failed to restore adequate blood flow. Heparin and antiplatelet treatment were provided for pharmacological management, but follow-up angiography 15 days later still revealed a poor result. This patient was ultimately treated with antiplatelet therapy in combination with warfarin treatment.

**Outcomes:**

Follow-up coronary angiography 15 months later showed a restored normal Thrombolysis In Myocardial Infarction grade (TIMI) 3 flow.

**Lessons:**

CAE-related infarct is often associated with high-burden thrombus formation. Long-term warfarin in combination with antiplatelet therapy may be a good alternative intervention to decrease thrombus burden and enhance blood flow.

## 1 Introduction

Coronary artery ectasia (CAE) is characterized by an abnormal dilatation of the coronary arteries. CAE is often associated with the presence of slow coronary flow, which may result in acute myocardial infarction (AMI), even without the total occlusion of the affected artery.^[1]^ Additionally, the management of CAE patients complicated by a large thrombus burden is a significant clinical challenge encountered during primary coronary intervention. Here, we report a case of CAE complicating an AMI, with a large thrombus burden that was resistant to repetitive aspiration thrombectomy.

## 2 Case report

Written informed consent was obtained, and institutional Ethics Committee of the Tianjin Medical University approved this case report.

**Figure 1:**
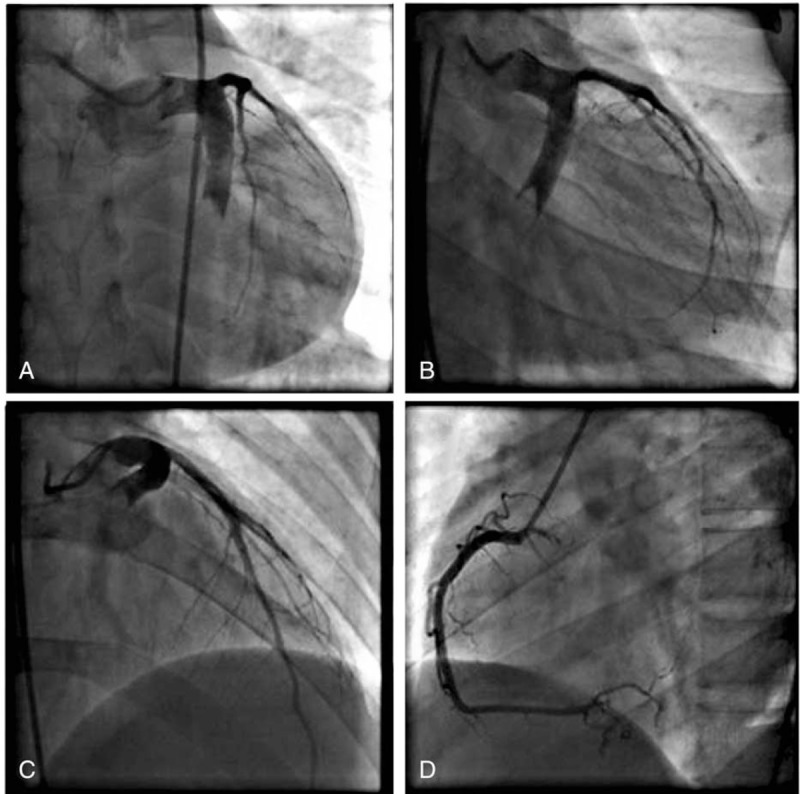
Coronary angiography images before thrombus aspirations. A–C, Ectasia of LM, and ectasia and total thrombotic occlusion of the distal LCX. The left anterior descending artery was normal. D, The right coronary artery was normal. LCX = left circumflex artery, LM = left main artery.

**Figure 2:**
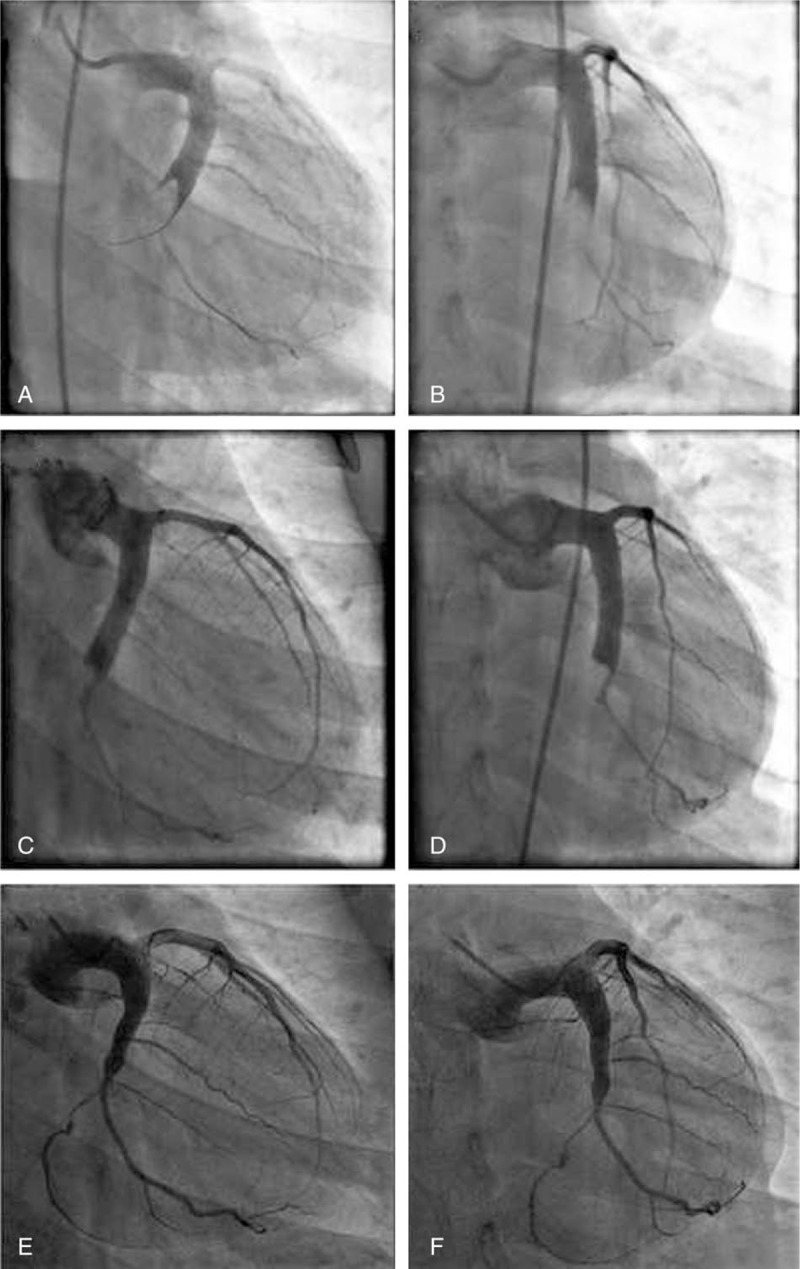
Angiographic images of the LCX, postthrombus aspirations in (A) right caudal view and (B) antero-posterior caudal view. After several attempted thrombus aspirations, only TIMI 2 flow was restored in the second obtuse marginal branch. The second coronary angiography images of the LCX 15 days after admission in (C) right caudal view and (D) antero-posterior caudal view. There was still an obstructive filling defect in the distal portion of the LCX. TIMI 3 flow was restored in the second obtuse marginal branch. The third coronary angiography images of the LCX 15 months after discharge in (E) right caudal view and (F) antero-posterior caudal view. TIMI 3 flow was restored in the LCX and almost complete resolution of the thrombus was noted. LCX = left circumflex artery, TIMI = thrombolysis in myocardial infarction grade.

**Figure 3:**
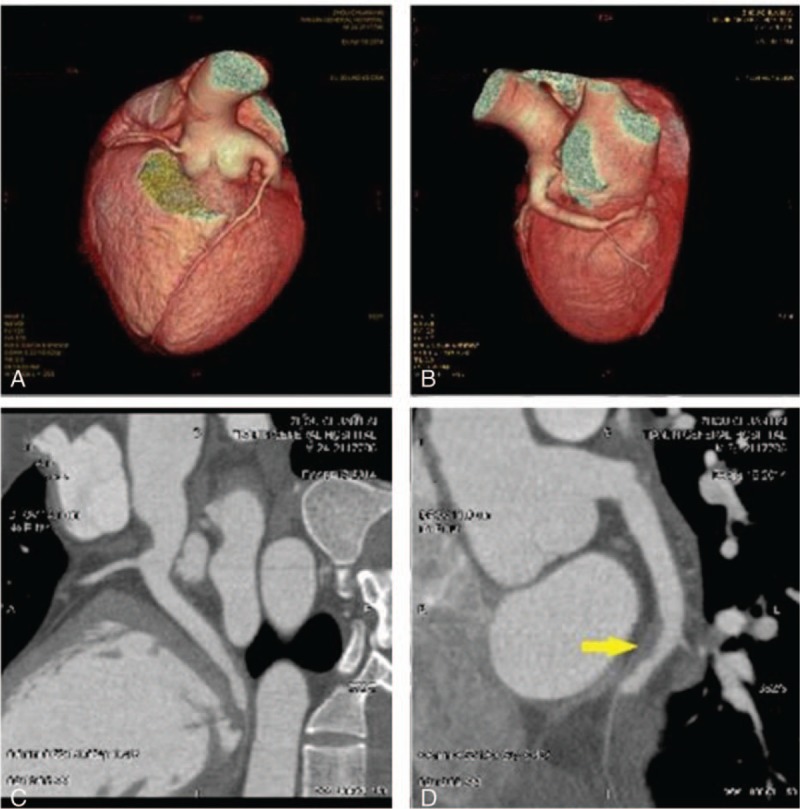
Sixty-four-slice coronary computed tomographic angiography images of the LCX 10 days after admission. A and B, Three-dimensional computed tomography reconstruction showing coronary artery ectasia involving the LM and LCX. C and D, A filling defect is shown in the distal portion of the LCX (arrow). LCX = left circumflex artery, LM = left main artery.

A 24-year-old male patient presented with severe, crushing chest pain 4 hours after the onset. His initial electrocardiogram (ECG) showed ST-segment elevation in leads II, III, aVF, V5, V6, and V7-V9. Upon physical examination, his heart rate was 75 bpm and arterial blood pressure was 130/80 mm Hg. Chest and heart examinations revealed nothing unusual. The troponin T level was elevated (0.567 ng/mL). Emergency coronary angiography showed ectasia of left main artery (LM), and ectasia and total thrombotic occlusion of the distal left circumflex artery (LCX) (Fig. 1A–C). There was a large thrombus burden within the CAE segment of the LCX (Fig. 1A–C). The left anterior descending artery (Fig. 1A–C) and right coronary artery (Fig. 1D) were normal. The decision to attempt primary percutaneous coronary intervention (PCI) on the LCX was based on an electrocardiogram and coronary angiogram. Due to the angiographical evidence of a heavy thrombus burden, we attempted PCI using repetitive coronary aspirations with a 6-French Export Catheter (Medtronic, Minneapolis, MN) for thrombus extraction. However, the lesion was resistant to repetitive aspirations and the blood flow was only partially restored in the second obtuse marginal branch (OM2) with Thrombolysis In Myocardial Infarction grade (TIMI) 2 flow (Fig. 2A and B). Consequently, the patient received tirofiban (10 μg/kg body weight) through guiding catheter after thrombus aspiration, and following 36 hours of intravenous infusion (0.15 μg/kg/min). Intracoronary thrombolysis was not performed in consideration of the potential bleeding risk. After considering that this patient suffered from a single-vessel lesion, was hemodynamically stable and his clinical symptoms had improved after PCI, emergency coronary artery bypass surgery was not performed. He instead received subcutaneous enoxaparin, oral aspirin, clopidogrel, atorvastatin, and ramipril. His chest pain was completely relieved following medical therapy. Eventually, his ECG showed ST-segment resolution. Coronary computed tomographic angiography 10 days after admission showed CAE involving the LM and LCX and confirmed the presence of an occlusive intraluminal thrombus in the distal portion of the LCX (Fig. 3). The patient underwent a second coronary angiography 15 days after admission. Coronary angiography showed that the ectasia remained and that there was an obstructive filling defect in the distal portion of the LCX. TIMI 3 flow was restored in OM2 (Fig. 2C and D). The patient was discharged on dual antiplatelet therapy. Oral anticoagulation was added in view of the spontaneous thrombus formation. Clopidogrel was stopped after 6 weeks, and the patient remained on long-term aspirin and warfarin. Follow-up coronary angiography was repeated 15 months later. The angiogram revealed that there was an almost complete resolution of the thrombus in the LCX, in which TIMI 3 flow was observed (Fig. 2E and F). Therefore, long-term oral anticoagulation with warfarin was prescribed for outpatient follow-up.

## 3 Discussion

CAE has been defined as localized or diffuse dilation of the coronary arteries more than 1.5 times the diameter of an adjacent healthy reference segment on coronary angiography.^[2]^ Atherosclerosis is considered to be the most frequent (50%) cause of CAE, whereas 20% to 30% cases of CAE are caused by congenital anomalies.^[3–5]^ Only 10% to 20% of cases of CAE have been reported resulting from inflammatory or connective tissue diseases.^[1,6]^ Our patient had neither clinical history nor laboratory tests results supporting the existence of vasculitis or atherosclerosis. Thus, the most probable cause of AMI in our patient was the CAE caused by congenital anomalies.

Clinical observations have demonstrated that thrombosis, embolization, and rupture of the involved segments are the leading causes of AMI and sudden death in patients with CAE.^[7,8]^ In recent years, optical coherence tomography (OCT) has emerged as the most accurate method for intracoronary evaluation. Owing to a resolution of 10 to 20 μm, OCT is more accurate than intravascular ultrasound (IVUS) and is useful for the evaluation and characterization of plaque features, both in stable and acute coronary artery disease.^[9]^ Unfortunately, due to the large size of the affected vessel, OCT was not feasible. IVUS could have been useful to better understand whether this case of AMI secondary to spontaneous thrombosis and without coronary plaque was caused by intimal inflammation, coronary embolism, or another factor. However, we could not use IVUS due to its unaffordability in our case. In the clinical setting of AMI, majority of CAE is positively related to high-burden thrombus formation and lower rate of successful reperfusion. When treated by primary percutaneous coronary intervention (pPCI), the existence of a massive intracoronary thrombus may lead to increased incidence of adverse outcomes.^[10–12]^ During balloon dilatation or stent implantation, the thrombus was crushed. This procedure could have subsequently caused the thrombus to embolize distally. The dislodged thrombus further activated platelets and this resulted in no reflow in the infarct-related artery.^[13]^ In our case, we did not perform a balloon dilatation because of concerns about the no-reflow phenomenon. The extensive thrombosis associated with CAE is generally accompanied by a reduced flow rate. Our case presented with acute inferior, posterior, and lateral myocardial infarction, which was caused by high-burden thrombus formation within the CAE. Similarly, in our case, the reperfusion therapy was not entirely successful and, due to the large vessel, a 6 Fr catheter was unable to remove all of the thrombus. Although a 7 Fr catheter, direct aspiration from the guiding catheter (or by a GuideLiner catheter with a “child in mother” coaxial system) or mechanical thrombus aspiration might have been more efficacious, they may also increase the risk of stroke linked to the procedure.^[14]^ Therefore, the therapeutic decision and appropriate management should be focused on how to reduce the heavy thrombus burden, which would consequently improve coronary reflow. From our experience, we strongly recommend a tailored strategy for each individual suffering from CAE-complicated AMI, which is dependent on clinical symptoms and angiographic findings, as well as the thrombus burden. If the patient's response to standard management is unsatisfactory, the early administration of warfarin may provide an alternative, in combination with antiplatelet medications. In our case, we used triple therapy (warfarin, aspirin, and clopidogrel) for 6 weeks. The clopidogrel was discontinued after 6 weeks and the patient remained on long-term aspirin and warfarin. After aspirin and warfarin treatment for 15 months, an outpatient follow-up showed that the patient was clinically stable, which was further confirmed by a follow-up coronary angiography that showed that a normal TIMI 3 flow was completely restored in the affected artery. Novel oral anticoagulant medications and antiplatelet P2Y_12_ receptor inhibitors have recently become available. However, no data suggest that use of these novel oral antithrombotics can improve the outcome of CAE patients complicated by a large thrombus burden.

## 4 Conclusions

In summary, CAE-related infarct is often associated with high-burden thrombus formation and has a significantly lower rate of successful reperfusion. Our experience has shown that in CAE patients presenting with AMI and evidence of a large thrombus burden during primary PCI, long-term oral anticoagulation treatment plus a tailored antiplatelet regimen may be an alternative option for those patients who do not respond to routine treatment. Further studies are needed to fully evaluate the clinical efficacy of this strategy in large patient samples.

## References

[1] Befeler B Aranda MJ Embi A Coronary artery aneurysms: study of the etiology, clinical course and effect on left ventricular function and prognosis. Am J Med 1977;62:597–607. 300567 10.1016/0002-9343(77)90423-5

[2] Swaye PS Fisher LD Litwin P Aneurysmal coronary artery disease. Circulation 1983;67:134–8. 6847792 10.1161/01.cir.67.1.134

[3] Hartnell GG Parnell BM Pridie RB Coronary artery ectasia. Its prevalence and clinical significance in 4993 patients. Br Heart J 1985;54:392–5. 4052280 10.1136/hrt.54.4.392 PMC481917

[4] Daoud AS Pankin D Tulgan H Aneurysms of the coronary artery. Report of ten cases and review of literature. Am J Cardiol 1963;11:228–37. 14025069 10.1016/0002-9149(63)90064-x

[5] Yetkin E Waltenberger J Novel insights into an old controversy: is coronary artery ectasia a variant of coronary atherosclerosis? Clin Res Cardiol 2007;96:331–9. 17453130 10.1007/s00392-007-0521-0 PMC2775118

[6] Mavrogeni S Coronary artery ectasia: from diagnosis to treatment. Hellenic J Cardiol 2010;51:158–63. 20378518

[7] Swanton RH Thomas ML Coltart DJ Coronary artery ectasia—a variant of occlusive coronary arteriosclerosis. Br Heart J 1978;40:393–400. 646906 10.1136/hrt.40.4.393 PMC482810

[8] Jang IK Lassila R Fuster V Atherogenesis and inflammation. Eur Heart J 1993;14 (suppl K):2–6. 8131783

[9] Iannaccone M Quadri G Taha S Prevalence and predictors of culprit plaque rupture at OCT in patients with coronary artery disease: a meta-analysis. Eur Heart J Cardiovasc Imaging 2015;17:1128–37. 26508517 10.1093/ehjci/jev283

[10] Otsuka M Minami S Hato K Acute myocardial infarction caused by thrombotic occlusion of a coronary aneurysm. Cathet Cardiovasc Diagn 1997;41:423–5. 9258490 10.1002/(sici)1097-0304(199708)41:4<423::aid-ccd19>3.0.co;2-m

[11] Kruger D Stierle U Herrmann G Exercise-induced myocardial ischemia in isolated coronary artery ectasias and aneurysms (“dilated coronopathy”). J Am Coll Cardiol 1999;34:1461–70. 10551693 10.1016/s0735-1097(99)00375-7

[12] Hokimoto S Saito T Noda K Relation between coronary thrombus and angiographic no-flow during primary angioplasty in patients with acute myocardial infarction. Jpn Circ J 1999;63:849–53. 10598889 10.1253/jcj.63.849

[13] Yip HK Wu CJ Chang HW Comparison of impact of primary percutaneous transluminal coronary angioplasty and primary stenting on short-term mortality in patients with cardiogenic shock and evaluation of prognostic determinants. Am J Cardiol 2001;87:1184–8. 11356395 10.1016/s0002-9149(01)01491-6

[14] Schiele F Ecarnot F Does thrombo-aspiration still have a place in the treatment of myocardial infarction? BMC Cardiovasc Disord 2016;16:97. 27206487 10.1186/s12872-016-0291-6 PMC4874010

